# Constructing Stable Sub/Surface Structure to Boost Superior Cyclabilities of Single‐Crystalline Ni‐Rich Cathode

**DOI:** 10.1002/advs.202510817

**Published:** 2025-08-30

**Authors:** Youqi Chu, Kai Wang, Changdong Chen, Dixing Ni, Qimeng Zhang, Hao Wang, Fan Peng, Anjie Lai, Gemeng Liang, Chenghao Yang

**Affiliations:** ^1^ Guangzhou Key Laboratory for Surface Chemistry of Energy Materials New Energy Research Institute School of Environment and Energy South China University of Technology Guangzhou 510006 P. R. China; ^2^ School of Chemical Engineering The University of Adelaide Adelaide SA 5000 Australia; ^3^ School of Advanced Materials Peking University, Shenzhen Graduate School Shenzhen Guangdong 518055 P. R. China; ^4^ High Performance Computing Department National Supercomputing Center in Shenzhen Shenzhen 518055 P. R. China

**Keywords:** electrochemical performance, LiNi_0.90_Co_0.05_Mn_0.05_O_2_, Lithium‐ion battery, single‐crystalline Ni‐rich cathode, thermodynamic stability

## Abstract

Under prolonged high‐voltage cycling, single‐crystalline Ni‐rich cathodes are prone to severe transition metal dissolution, irreversible phase transformations, and reduced structural stability, which significantly hinder their practical application. Hence, a dual‐modification strategy is proposed and implemented for single‐crystalline LiNi_0.90_Co_0.05_Mn_0.05_O_2_ (SCNCM90) cathodes by introducing Al/B gradient co‐doping and an Al_5_BO_9_ surface coating to mitigate anisotropic structural changes. The subsurface Al/B gradient doping induces a lithium‐rich vacancy disordered structure, which effectively suppresses the H2‐H3 phase transition, while suppressing lattice strain and mechanical degradation. In parallel, the chemically stable Al_5_BO_9_ surface coating significantly mitigates harmful electrode‐electrolyte interfacial reactions, thereby enhancing both structural and electrochemical stability under high‐voltage conditions. Electrochemical tests reveal that the Al/B co‐modified SCNCM90 electrode exhibits markedly improved performance, achieving 95.83% capacity retention after 200 cycles at 4.5 V and maintaining 89.6% retention at 1C after 1000 cycles in pouch‐type full cells within 3–4.25 V. Moreover, the modified electrode demonstrates superior lithium‐ion diffusion kinetics and enhanced thermodynamic stability during cycling. This effective dual‐modification strategy offers a promising pathway to improve the structural robustness and electrochemical durability of single‐crystalline Ni‐rich cathodes, thus accelerating their adoption in next‐generation high‐energy lithium‐ion batteries.

## Graphical abstract

A dual‐modification strategy combining gradient Al/B co‐doping and a robust Al_5_BO_9_ surface coating in this work enables synergistic bulk and surface stabilization in single‐crystalline LiNi_0.90_Co_0.05_Mn_0.05_O_2_ for lithium‐ion batteries. The engineered cathode exhibits 89.6% capacity retention over 1000 cycles in pouch full cells, among the best performances reported for single‐crystalline Ni‐rich cathodes.

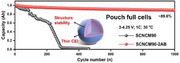

## 1 Introduction

Single‐crystalline Ni‐rich layered oxide LiNi_0.90_Co_0.05_Mn_0.05_O_2_ (SCNCM90) has emerged as one of the most promising cathode materials for lithium‐ion batteries (LIBs) in long‐range electric vehicles, owing to its high specific discharge capacity (> 200 mAh g^−1^), cost‐effectiveness, and relatively low environmental impact.^[^ ^1^, ^2^ ^]^ However, ultra‐high nickel content (Ni > 90%) introduces inherent structural instabilities at both the bulk and interface levels, severely limiting long‐term cycling performance.^[^ ^3^ ^]^ Specifically, at high states of charge, the abrupt anisotropic lattice contraction along the *c*‐axis caused by the H2→H3 phase transition induces substantial mechanical stress, which is the primary driver of microcrack formation during prolonged cycling.^[^ ^4^, ^5^ ^]^ These microcracks facilitate electrolyte penetration into the interior of active particles, accelerating parasitic reactions at the internal electrode‐electrolyte interface.^[^ ^6^ ^]^ This, in turn, promotes the formation of detrimental NiO‐like phases and a continuous increase in interfacial impedance.^[^ ^7^ ^]^ Additionally, hydrofluoric acid (HF), generated from electrolyte decomposition, aggressively corrodes the particle surface and leads to transition metal (TM) dissolution.^[^ ^8^, ^9^ ^]^ Compounding these issues, oxygen release under high delithiation and elevated voltages exacerbates electrolyte decomposition while inducing severe lattice strain, structural collapse, and chemical‐mechanical degradation, all of which pose significant safety concerns.^[^ ^10^, ^11^ ^]^ Consequently, enhancing the cycling/structure stability of Ni‐rich cathodes under high‐voltage conditions and enabling their large‐scale application necessitates addressing these fundamental challenges through targeted material design and engineering strategies.

To mitigate these challenges and enhance the electrochemical performance of Ni‐rich cathodes, various approaches have been explored, including element doping, microstructural engineering, and the development of single‐crystalline architectures.^[^ ^12^, ^13^, ^14^, ^15^ ^]^ However, these conventional strategies often fall short in fundamentally addressing the intrinsic causes of structural degradation, such as lattice displacement and anisotropic lattice evolution.^[^ ^16^, ^17^ ^]^ Thus, the development of an effective strategy to dissipate internal strain, suppress microcrack formation, and block electrolyte intrusion is critical for achieving durable structural integrity.^[^ ^18^ ^]^ Inspired by the inherent robustness of cation‐disordered structures, we propose a novel design strategy that integrates a subsurface cation‐disordered architecture with a chemically stable surface coating. Specifically, a thin cation‐disordered layer was introduced at the surface, mitigating the detrimental effects of electrode‐electrolyte interfacial reactions while suppressing lattice strain and mechanical degradation, all without compromising capacity or kinetic performance. Consequently, the subsurface cation‐disordered structure effectively curbs oxygen evolution, impedes the diffusion of oxygen vacancies into the bulk phase, and enhances overall structural stability. Hence, constructing stable sub/surface structure aims to prevent structural collapse and inhibit transition metal migration during deep delithiation, thereby addressing the root causes of failure in Ni‐rich cathodes.

In this work, we propose and successfully demonstrate a dual‐modification strategy for single‐crystalline Ni‐rich LiNi_0.90_Co_0.05_Mn_0.05_O_2_ (SCNCM90) cathodes that combines Al/B gradient co‐doping with an Al_5_BO_9_ surface coating to overcome structural instability under high‐voltage cycling. The Al/B gradient co‐doping introduced a lithium‐rich, vacancy‐containing disordered subsurface structure, which effectively mitigated lattice strain and mechanical degradation while maintaining excellent capacity retention and lithium‐ion kinetics. Meanwhile, the Al_5_BO_9_ coating provided chemical protection by suppressing detrimental electrode‐electrolyte interface reactions, thereby further improving structural and electrochemical stability. Electrochemical evaluations revealed that the Al/B co‐modified SCNCM90 cathode exhibited outstanding performance, achieving 95.83% capacity retention after 200 cycles at 4.5 V, while exhibiting superior lithium‐ion diffusion kinetics and excellent thermodynamic stability throughout cycling. In a pouch‐type full‐cell, the SCNCM90‐2AB cathode retains 89.6% of its capacity after 1000 cycles at 1C. These results underscore the effectiveness of the proposed dual‐modification strategy in resolving structural degradation issues in Ni‐rich cathodes and offer a promising pathway for the large‐scale application of high‐energy‐density lithium‐ion batteries.

## 2 Results and Discussion

**Figure 1:**
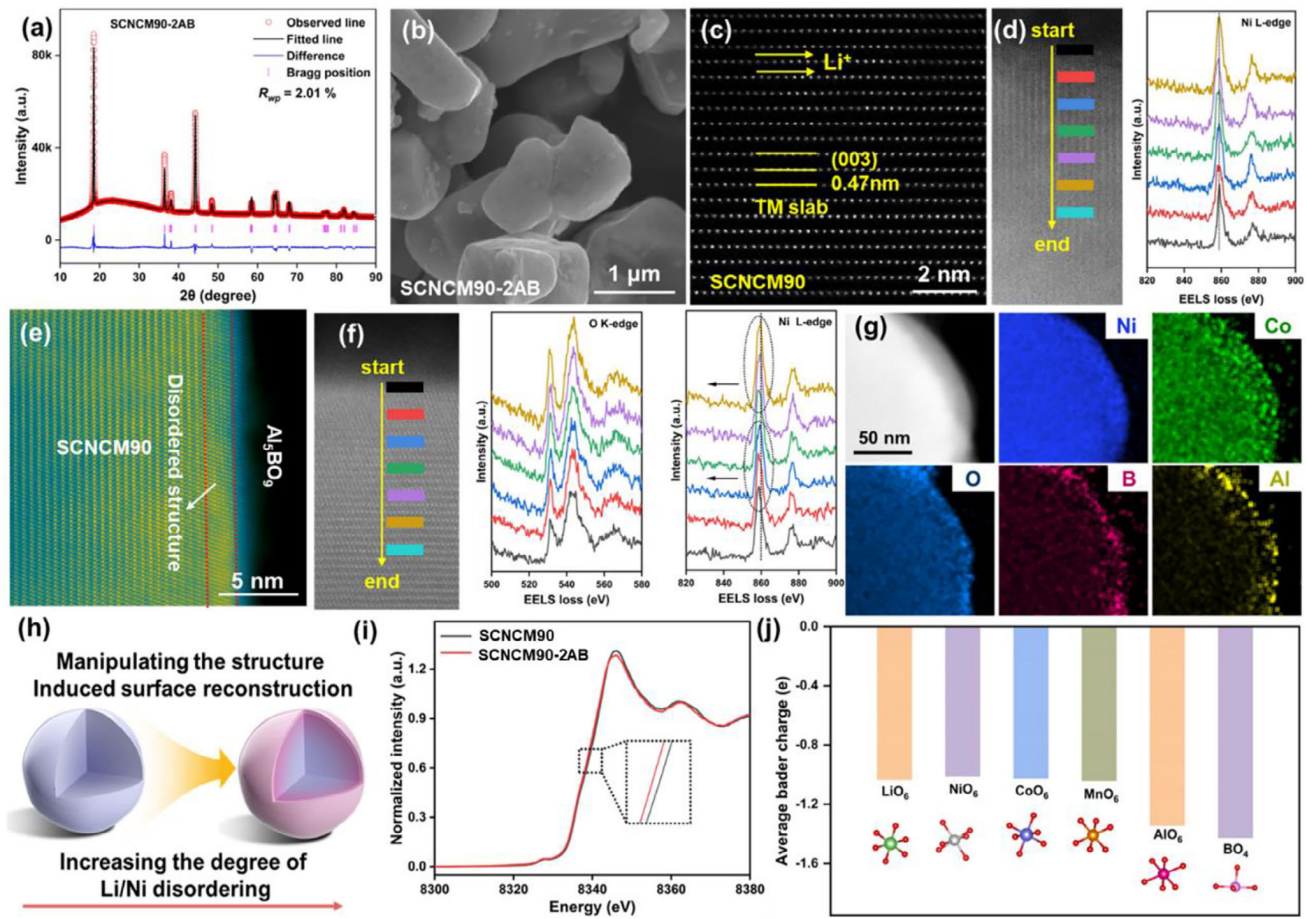
a) The Rietveld refinement results of SCNCM90‐2AB. b) SEM images of SCNCM90‐2AB. HAADF‐STEM images of c) SCNCM90 and e) SCNCM90‐2AB. d,f) EELS spectra of the Ni L‐edges and O K‐edges extracted from the surface to the inside region of SCNCM90 and SCNCM90‐2AB. g) HRTEM‐EDS of SCNCM90‐2AB. h) Schematic illustration of the structural evolution of the SCNCM90 surface. i) Ni K‐edge XANES spectra of SCNCM90 and SCNCM90‐2AB. j) Average Bader charge of the O atoms in the MO_6_ octahedron (M = Li/Ni/Co/Mn/Al) and BO_4_ tetrahedron.

The Al/B co‐modified SCNCM90 (denoted as SCNCM90‐*x*AB, where *x* represents the concentration of Al and B) was synthesized via a solution‐based coating process followed by thermal treatment. Specifically, SCNCM90 powder was dispersed in an ethanol solution containing stoichiometric amounts of Al(NO_3_)_3_·9H_2_O and H_3_BO_3_, and the mixture was heated and stirred continuously at 70 °C. The resulting slurry was subsequently dried and calcined at 600 °C in air (see Methods and Figure S1, Supporting Information). Inductively coupled plasma optical emission spectroscopy (ICP‐OES) confirmed that the elemental composition of Ni, Co, Mn, Al, and B in the final materials closely matched the designed stoichiometry (Table S1, Supporting Information). X‐ray diffraction (XRD) analysis reveals that both pristine SCNCM90 and the modified SCNCM90‐2AB maintain a well‐defined layered structure, with only a slight increase in Li/Ni cation mixing from 1.16% to 1.21% after modification (**Figure** **1**a; Figure S2; Rietveld refinement details in Table S2, Supporting Information). Scanning electron microscopy (SEM) images show that the single‐crystal morphology and particle size (2–5 µm) were preserved after surface modification (Figure 1b; Figure S3, Supporting Information). Energy‐dispersive X‐ray spectroscopy (EDS) mapping confirmed the homogeneous distribution of Ni, Co, Mn, O, Al, and B elements across the SCNCM90 surface (Figure S4, Supporting Information).

High‐angle annular dark‐field scanning transmission electron microscopy (HAADF‐STEM) revealed a lattice spacing of 0.47 nm, corresponding to the (003) plane of the layered SCNCM90 structure (Figure 1c).^[^ ^19^ ^]^ Transmission electron microscopy (TEM) further confirmed the formation of a thin, uniform surface coating with poor crystallinity (Figure S5, Supporting Information). To validate the formation of the Al_5_BO_9_ phase, a control experiment was conducted using Al(NO_3_)_3_·9H_2_O and H_3_BO_3_ under identical calcination conditions. The resulting powder exhibited XRD peaks consistent with the crystalline phase of Al_5_BO_9_ (PDF#97‐002‐0172) (Figure S6, Supporting Information), confirming the Al_5_BO_9_ formation on the SCNCM90 particle surface.^[^ ^20^ ^]^ The Al_5_BO_9_ nanocoating was observed to be in intimate contact with the SCNCM90 surface, without any interfacial gaps, suggesting strong interfacial adhesion. Notably, although the bulk structure retained a well‐ordered layered framework, a slight lattice reconstruction was detected in the subsurface region (≈3 nm depth), characterized by a Li/Ni disordered arrangement (highlighted in Figure 1e). Spatially resolved STEM‐EELS analysis was used to investigate the chemical environment transition from the surface to the bulk. Line‐scan EELS spectra (Figure 1d,f) show a gradual shift in the Ni L‐edges of SCNCM90‐2AB (Figure 1f), indicating a surface Ni^2+^‐rich environment. This Ni^2+^‐enriched surface has been previously associated with improved structural and interfacial stability.^[^ ^21^ ^]^ Additionally, the O K‐edges spectrum exhibits a weak pre‐edge peak (Figure 1f; Figure S7, Supporting Information), suggesting the presence of surface oxygen vacancies in both samples. EDS elemental mapping further verified the homogeneous distribution of transition metals and oxygen alongside the surface enrichment of Al and B (Figure 1g).

The structural evolution induced by Al/B modification was illustrated through a schematic, showing the subsurface lattice reconstruction via increased Li/Ni disordering (Figure 1h). X‐ray absorption near‐edge structure (XANES) analysis of the Ni K‐edge revealed a shift to lower energy in SCNCM90‐2AB, indicating partial reduction of Ni^3+^ to Ni^2+^, consistent with the charge compensation caused by Al^3+^ and B^3+^ incorporation (Figure 1i). Fourier‐transform extended X‐ray absorption fine structure (FT‐EXAFS) and wavelet transform (WT) analysis (Figure S8, Supporting Information) further show a narrower Ni─O shell in SCNCM90‐2AB compared to pristine SCNCM90, indicative of stronger Ni 3d‐O 2p hybridization. As a result, SCNCM90‐2AB exhibits a thicker LiO_6_ interlayer, stronger Ni─O bonding, and improved electrochemical stability.^[^ ^22^ ^]^ Soft X‐ray absorption spectroscopy (sXAS) was employed to investigate the valence states and structural characteristics of surface elements (Figure S9, Supporting Information). The analysis revealed that the valence state of Ni exhibits a positive correlation with the B/A ratio (where A and B represent the low‐energy and high‐energy states in the Ni L_3_‐edge, respectively); an increase in this ratio corresponds to an elevated content of Ni^3+^ in the bulk. The B/A ratio significantly decreased following Al^3+^/B^3+^ dual‐modification, declining from 0.71 to 0.68. The reduction in the B/A ratio suggests a decrease in the Li^+^/Ni^2+^ mixture, which aligns with the results obtained from Rietveld refinement. X‐ray photoelectron spectroscopy (XPS) was further utilized to quantify the relative contents of Ni^2+^ and Ni^3+^ on the surfaces of SCNCM90 and SCNCM90‐2AB (Figure S10, Supporting Information). In the Ni 2p_3/2_ spectrum, the characteristic peaks at 852.7 and 854.1 eV were assigned to Ni^2+^ and Ni^3+^, respectively. The results revealing a higher Ni^2+^/Ni^3+^ ratio in SCNCM90‐2AB compared to the SCNCM90, demonstrate an increased degree of Li^+^/Ni^2+^ disorder, consistent with the XANES results. Bader charge analysis indicates that Al atoms preferentially substitute TM, while B atoms occupy tetrahedral interstitial positions. The average charge on O atoms in AlO_6_ and BO_4_ units was ≈0.3 eV lower than in LiO_6_, NiO_6_, CoO_6_, and MnO_6_ environments, reflecting enhanced electron density on oxygen and a more robust anionic framework (Figure 1j).^[^ ^23^ ^]^ Charge density distribution analysis further confirmed the enhanced covalency of Al/B─O bonds in SCNCM90‐2AB, which introduces additional electron density around oxygen atoms (Figure S11, Supporting Information), reinforcing the oxygen lattice and mitigating oxygen loss during high voltage cycling. Together, these findings demonstrate that Al/B co‐doping not only stabilizes the transition metal layer but also strengthens the oxygen framework via electronic modulation, offering a comprehensive solution for enhancing the structural durability of Ni‐rich cathodes.

**Figure 2:**
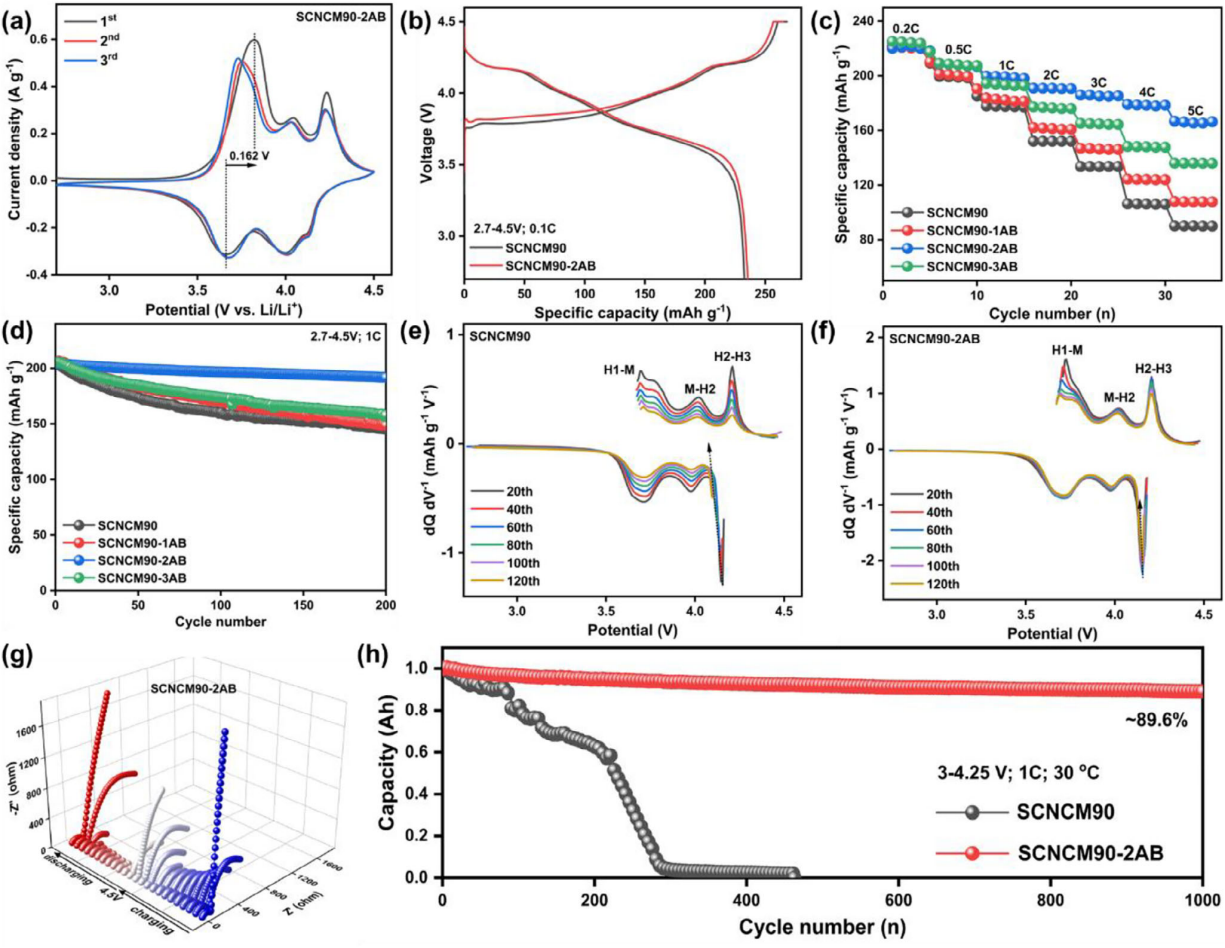
a) The CV curves of the first three cycles scanned at 0.1 mV s^−1^ of SCNCM90‐2AB. b) Initial charge/discharge curves within 2.7–4.5 V for SCNCM90 and SCNCM90‐2AB. c,d) Rate performance and cycle performance at 1C for all samples. e,f) The dQ dV^−1^ curves by differentiating charge/discharge curves at different cycle numbers for SCNCM90 and SCNCM90‐2AB. g) In situ Nyquist plots of SCNCM90‐2AB in the initial cycle. h) Cycle stability at 1C of SCNCM90/SCNCM90‐2AB||graphite pouch‐type full cells within 3–4.25 V.

Given the critical role of surface engineering in enhancing the structural and electrochemical stability of high‐energy cathodes, we conducted a comprehensive evaluation of the battery performance of Al/B co‐modified SCNCM90 samples. Cyclic voltammetry (CV) analysis reveals typical electrochemical behavior for Ni‐rich materials in both pristine and modified cathodes. Notably, the pristine SCNCM90 exhibits a significant potential difference (ΔV = 0.421 V, Figure S12, Supporting Information), whereas the SCNCM90‐2AB cathode shows a much smaller ΔV of 0.162 V (**Figure** **2**a), indicating reduced electrode polarization, enhanced interfacial stability, and improved structural reversibility. The initial charge/discharge profiles of SCNCM90 and SCNCM90‐2AB, measured between 2.7 and 4.5 V at 0.1C, are presented in Figure 2b. The SCNCM90‐2AB cathode delivers a specific discharge capacity of 235.58 mAh g^−1^ with an initial coulombic efficiency (ICE) of 89.01%, compared to the SCNCM90 (232.73 mAh g^−1^ and 86.79% ICE). These results suggest that Al/B co‐modification effectively reduces irreversible capacity loss and enhances lattice oxygen stability. Under high‐rate conditions (5C), the SCNCM90‐2AB cathode maintains a capacity of 165.37 mAh g^−1^, significantly outperforming its unmodified counterpart (90.11 mAh g^−1^) (Figure 2c), which is attributed to the improved Li^+^ diffusion kinetics facilitated by the dual‐modification strategy. Galvanostatic intermittent titration technique (GITT) analysis further validated the enhanced lithium‐ion transport in SCNCM90‐2AB after 50 cycles (Figure S13, Supporting Information).

Meanwhile, the long‐term cycling tests at 1C in a half‐cell configuration identify SCNCM90‐2AB as the optimal cathode, with the highest capacity retention of 93.89% after 200 cycles due to the structural benefits of surface modification (Figure 2d). Differential capacity (dQ dV^−1^) analysis performed every 20 cycles (Figure 2e,f) reveals characteristic phase transitions in both cathodes, notably the H1‐M, M‐H2, H2‐H3 transitions. The dQ dV^−1^ peaks of pristine SCNCM90 shift progressively to lower voltages and diminish in intensity, indicative of irreversible phase degradation, while the SCNCM90‐2AB cathode maintains stable peak positions and intensities, underscoring its superior structural stability and reversibility during prolonged cycling.^[^ ^24^, ^25^ ^]^ The electrochemical impedance Nyquist plot after 100 cycles is shown in Figure S14 (Supporting Information). The results indicate that the Al/B co‐modification helps to form a thinner and more stable CEI film and reduces parasitic reactions. In situ galvanostatic electrochemical impedance spectroscopy (EIS) was employed to monitor the impedance evolution of both cathodes during the initial cycle (Figure 2g; Figure S15, Supporting Information). Nyquist plots reveal that the charge transfer resistance (R_ct_) of pristine SCNCM90 increases dramatically from ≈250 to 2000 Ω as the voltage rises from open circuit voltage (OCV) to 4.5 V versus Li/Li⁺. In contrast, SCNCM90‐2AB exhibits only a moderate increase in R_ct_ from 20 to 1600 Ω, with minimal impedance changes in the low‐frequency region, confirming its superior interfacial diffusion kinetics. To assess practical applicability, SCNCM90‐2AB was further evaluated in pouch‐type full cells operating at 3.0–4.25 V. After 1000 cycles at 1C, the Al/B dual‐modified cathode retains 89.6% of its initial capacity, significantly outperforming the pristine material (SCNCM90) (Figure 2h). These results comprehensively demonstrate the effectiveness of Al/B co‐modification in improving both the structural and electrochemical stability of Ni‐rich cathodes, establishing SCNCM90‐2AB as a highly promising candidate for high‐energy lithium‐ion battery applications.

**Figure 3:**
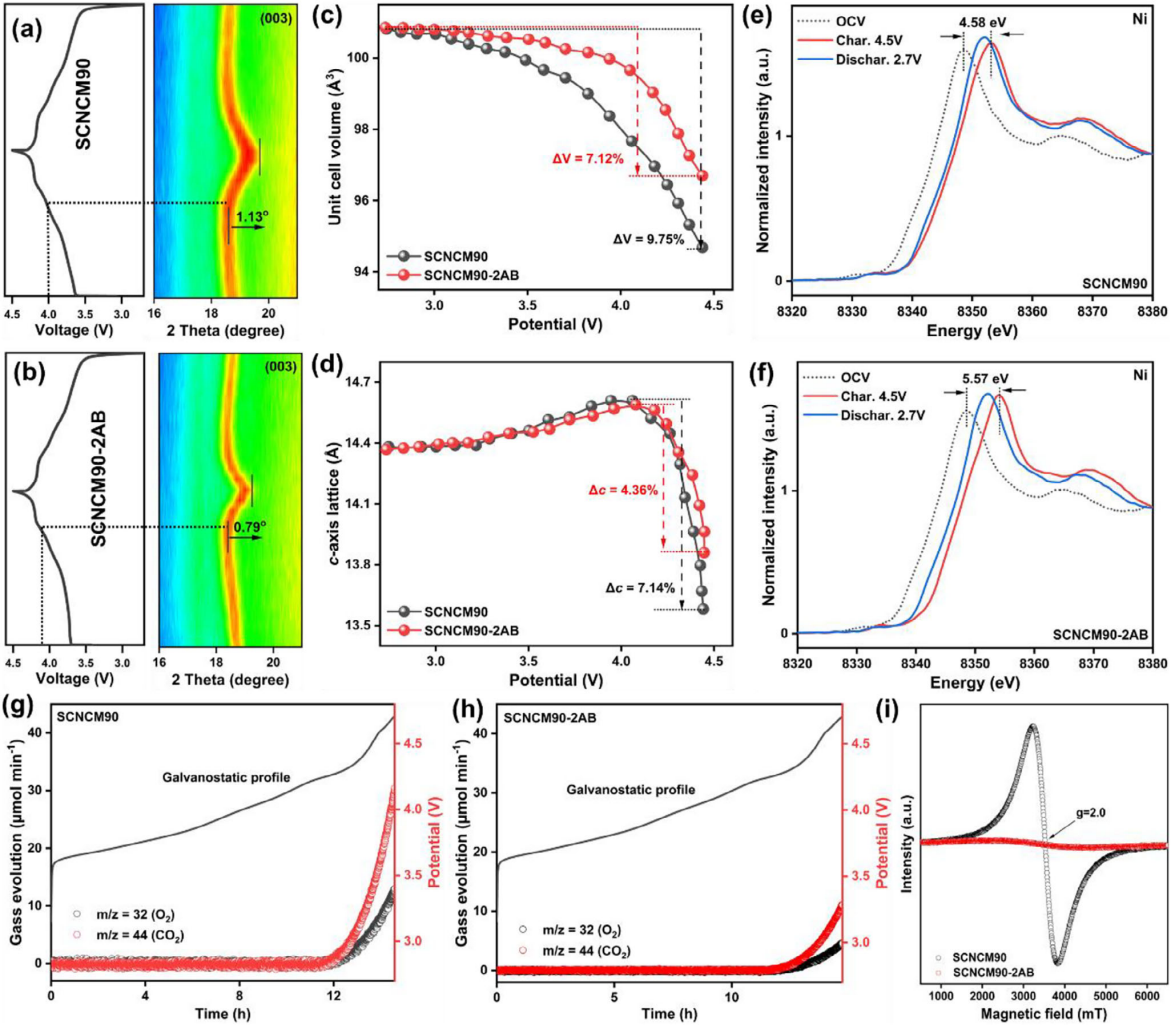
In situ XRD of a,b) (003) peak, c) lattice parameter *c*, and d) unit cell volume variation of SCNCM90 and SCNCM90‐2AB during the initial cycle. e,f) The change of Ni‐K edge XANES spectrum of SCNCM90 and SCNCM90‐2AB during the initial cycle. g,h) In situ DEMS of O_2_ and CO_2_ gas evolution for SCNCM90 and SCNCM90‐2AB during the initial charging process. i) EPR spectra of the SCNCM90 and SCNCM90‐2AB after 100 cycles.

To further quantify the phase transition behavior of the cathodes, in situ XRD analysis was conducted during the first charge process (2.7–4.5 V) at 0.1C (**Figure** **3**a,b). Lattice parameters (*c*, V) were derived from Rietveld refinement of the XRD spectra (Figure 3c). For the pristine SCNCM90 electrode, the (003) peak reflection gradually shifted to a lower angle as the voltage increased. This shift was attributed to phase transitions occurring at ≈3.65 V (hexagonal to monoclinic, H1→M) and at ≈4.02 V (monoclinic to hexagonal, M→H2). The (003) reflection peak shifted from 18.72° to 18.59°, corresponding to the initial expansion of the *c*‐axis lattice parameter. At ≈4.2 V, the (003) peak began to shift to a higher angle due to the contraction of the *c*‐axis lattice parameter during the hexagonal‐to‐hexagonal (H2→H3) phase transition, with a larger angular displacement (19.72°). This shift to a higher angle indicates a significant contraction of the *c*‐axis lattice parameter during the H2‐H3 phase transition. Conversely, the SCNCM90‐2AB electrode displayed a similar initial shift from 18.72° to 18.41°, but the subsequent detrimental contraction was markedly reduced, with the peak shifting only to 19.20°. This observation further confirms that the Al/B co‐modification inhibited the contraction of the *c*‐axis lattice, corresponding to the improved reversibility of the H2→H3 phase transition in SCNCM90‐2AB. However, compared to SCNCM90, SCNCM90‐2AB exhibited a voltage lag in the H2→H3 phase transition, suggesting that the Al/B dual modification enhances the TM‐O bond strength and suppresses the ordering of lithium vacancies, thereby delaying or weakening the H2‐H3 phase transition. Additionally, the surface Al_5_BO_9_ coating inhibited interfacial side reactions and delayed internal phase transitions, further enhancing the structural stability of the SCNCM83.^[^ ^26^ ^]^ These results indicate the Al/B co‐modification strategy effectively suppresses the *c*‐axis lattice contraction (Δ*c*, 4.36% for SCNCM90‐2AB vs 7.14% for SCNCM90). Consequently, the unit cell volume change (ΔV) (calculated using the formula V = *a* ^2^_*_ c_*_sin(π/3)) was significantly reduced in SCNCM90‐2AB (7.12%) than in SCNCM90 (9.75%). These findings confirm that Al/B dual modification mitigates the lattice deformation and helps preserve particle integrity during cycling.

Complementary XAFS analysis was employed to investigate the evolution of the Ni electronic structure during cycles (Figure 3d,e). During discharge, SCNCM90‐2AB experiences a larger peak position shift of 5.57 eV (Figure 3e) than that of SCNCM90 (4.58 eV, Figure 3d), which could be ascribed to the lower average valence of Ni caused by more Ni^2+^ observed in our XPS results. When discharging, a minor shift in the curves is observed in SCNCM90, indicating the insufficient Ni reduction during lithiation, which could result from the structure failure caused by the significant lattice contraction, whilst this phenomenon is absent in SCNCM90‐2AB.^[^ ^27^ ^]^ These results collectively highlight the role of Al/B co‐modification in stabilizing the cathode structure and improving electrochemical performance by mitigating lattice strain and suppressing detrimental side reactions. To further verify the improved lattice oxygen stability, we conducted in situ differential electrochemical mass spectrometry (DEMS) measurements during the first cycle (Figure 3f,g). The SCNCM90‐2AB electrode exhibits significantly reduced gas evolution (O_2_ and CO_2_) compared to pristine SCNCM90, confirming that Al/B co‐modification effectively inhibits lattice oxygen release.^[^ ^28^ ^]^ To further elucidate the lattice oxygen stability, electron paramagnetic resonance (EPR) measurements were also carried out on both electrodes after 100 cycles (2.7–4.5 V), with SCNCM90‐2AB showing much lower signal intensity (Figure 3i), further validating its enhanced oxygen stability.

**Figure 4:**
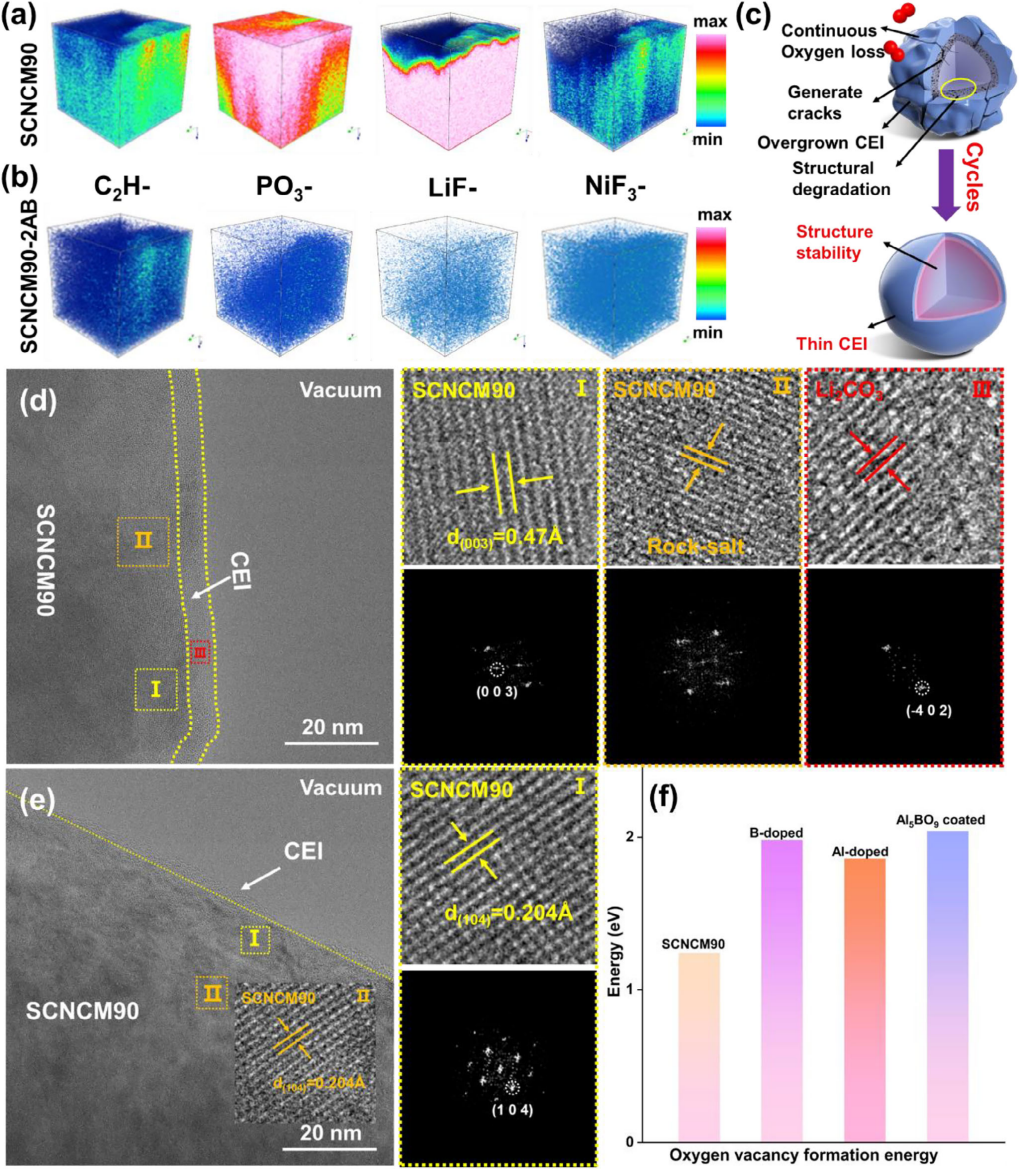
a,b) The 3D reconstruction of C_2_H‐, PO_3_‐, LiF_2_‐, and NiF_3_‐ fragments at the SCNCM90 and SCNCM90‐2AB cathodes surface after 100 cycles. c) Schematic diagrams of CEI grown for SCNCM90 and SCNCM90‐2AB electrodes during prolonged cycling. d,e) Cryo‐TEM images of SCNCM90 and SCNCM90‐2AB cathode surface after 100 cycles. f) The surface oxygen vacancy formation energy of SCNCM90, B‐doped, Al‐doped, and Al_5_BO_9_‐coated SCNCM90.

During electrochemical cycling, especially under high‐voltage charging, the Fermi energy level of the cathode approaches the highest occupied molecular orbital (HOMO) of the electrolyte, leading to an increase in hole concentration and promoting electrolyte oxidation. Simultaneously, dissolved transition metals (TMs) can react with electrolyte components to form metal fluorides. These processes contribute to the formation of a thick and complex cathode‐electrolyte interphase (CEI) layer, which hinders lithium‐ion transport and degrades electrochemical performance.^[^ ^29^, ^30^ ^]^ To analyze the chemical composition of the cathode surface after long‐term cycling, XPS was performed on pristine SCNCM90 and SCNCM90‐2AB electrodes after 200 cycles (Figure S16, Supporting Information). The XPS results show that the LiF signal on the SCNCM90‐2AB surface is significantly weaker than that of pristine SCNCM90, indicating that the Al/B co‐modification effectively suppresses interfacial side reactions. Additionally, inductively coupled plasma optical emission spectroscopy (ICP‐OES) was employed to quantify the dissolution of transition metals in the electrolyte after 200 cycles (Table S3, Supporting Information). The results demonstrated that SCNCM90‐2AB exhibits substantially lower TM dissolution than the unmodified SCNCM90, further confirming the improved interfacial stability enabled by the dual‐modification strategy. To further examine chemical evolution at the cathode‐electrolyte interface, time‐of‐flight secondary ion mass spectrometry (TOF‐SIMS) was conducted. As shown in **Figure** **4**a,b, the surface of pristine SCNCM90 exhibits higher intensities of C_2_H‐ and PO_3_‐ fragments, indicating severe electrolyte decomposition and the formation of a thicker CEI. In contrast, SCNCM90‐2AB shows reduced levels of LiF_2−_ and NiF_3−_ species after 100 cycles, suggesting enhanced resistance to acidic byproducts, such as HF. These observations point out a thinner CEI layer and lower fluoride content on SCNCM90‐2AB, which in turn facilitates better Li^+^ interfacial transport. A schematic diagram (Figure 4c) illustrates the long‐term evolution of SCNCM90 and SCNCM90‐2AB during cycling, highlighting the growth of CEI, lithium impurity accumulation, and oxygen loss. These structural changes contribute to degradation in pristine SCNCM90, while SCNCM90‐2AB maintains structural integrity.

Cryogenic transmission electron microscopy (cryo‐TEM) was used to examine the CEI structures after 100 cycles (Figure 4d,e). The cryo‐TEM images and corresponding fast Fourier transform (FFT) patterns reveal that the CEI on SCNCM90 primarily consists of a thick amorphous Li_2_CO_3_ layer. Weak diffraction rings corresponding to the (‐402) plane of Li_2_CO_3_ (2.56 Å) and the (003) plane of SCNCM90 (4.73 Å) were observed, with no detectable crystalline LiF. Furthermore, clear phase transition features were present on the SCNCM90 surface, indicating structural instability. In contrast, the CEI on SCNCM90‐2AB was significantly thinner and exhibited no discernible phase transitions. To better understand the origin of the observed stability, density functional theory (DFT)calculations were performed to evaluate the oxygen vacancy formation energy (Figure 4f). The SCNCM90‐2AB exhibits a higher oxygen vacancy formation energy than SCNCM90, attributed to the protective role of the Al/B dual modification, which shields the active material from electrolyte‐induced corrosion and mitigates Ni^4+^‐driven oxidation. Moreover, the stronger bond strengths of Al─O (607 kJ mol^−1^) and B─O (809 kJ mol^−1^), relative to Ni─O (391.6 kJ mol^−1^), further contribute to the stabilization of lattice oxygen. Collectively, these results demonstrate that the Al/B dual‐modification strategy effectively suppresses interfacial side reactions, reduces CEI thickness, inhibits oxygen loss, and enhances both lattice and interfacial chemical stability. The combination of experimental and theoretical evidence highlights the crucial role of Al/B co‐modification in enhancing the structural integrity and electrochemical performance of high‐Ni cathode materials.

**Figure 5:**
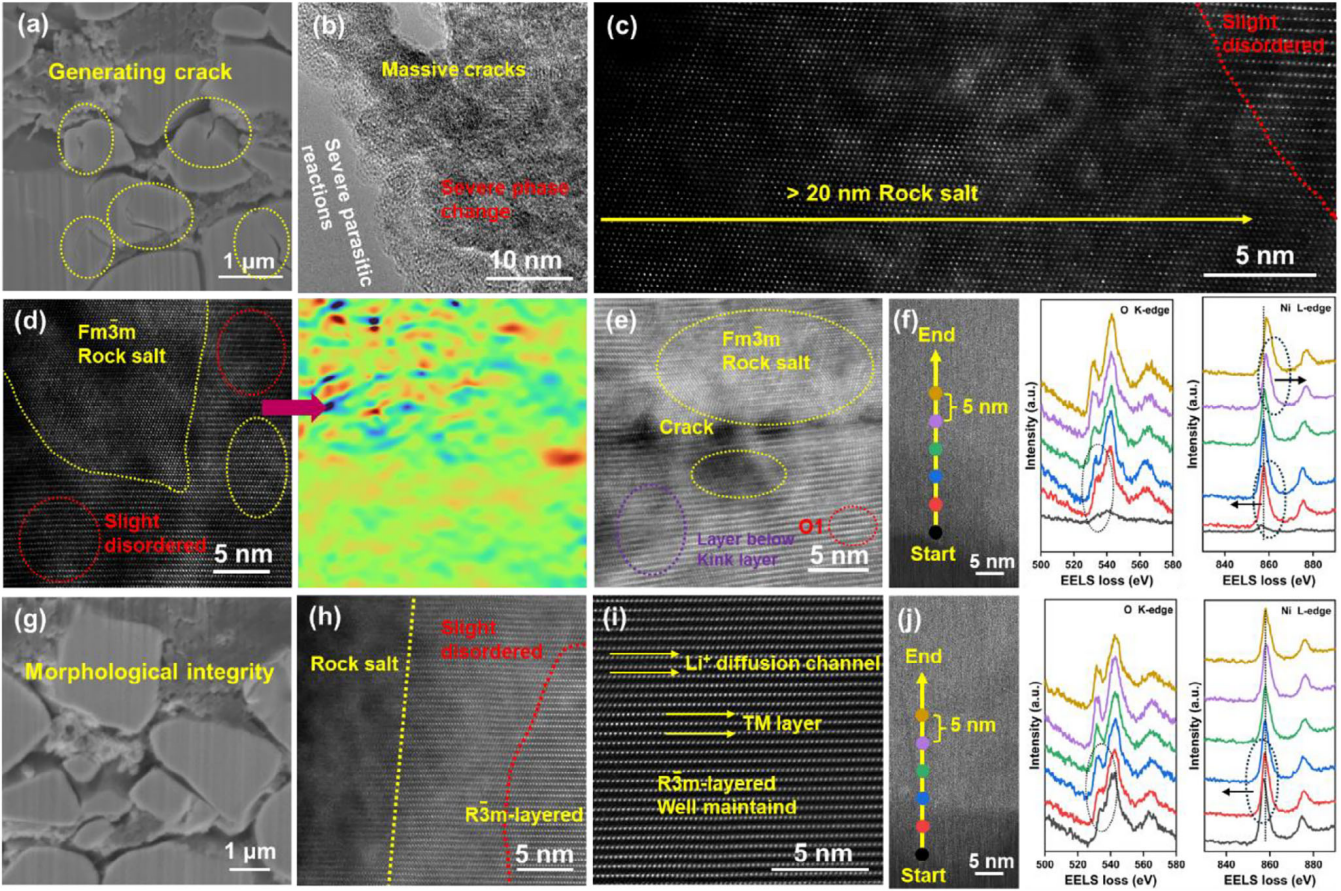
FIB‐SEM images of a) SCNCM90 and g) SCNCM90‐2AB cathodes after 200 cycles. b) HRTEM images of SCNCM90 cathodes after 200 cycles. c–e) Cross‐sectional low magnification HADDF‐STEM image of SCNCM90 and the corresponding ε_xx_ strain map were obtained by performing GPA patterns after 200 cycles. h,i) Cross‐sectional low magnification HADDF‐STEM image of SCNCM90‐2AB after 200 cycles. EELS spectra for f) SCNCM90 and j) SCNCM90‐2AB of O K‐edges, Ni L‐edges extracted from the surface to the inside region with colored line.

To elucidate the mechanism behind enhanced cycle stability, we performed comprehensive microstructure characterization of cycled cathodes. SEM revealed that the SCNCM90 single‐crystalline particles developed numerous microcracks after 200 cycles (**Figure** **5**a), resulting from uneven stress caused by lithium ion concentration gradients within the micrometer‐sized particles. In marked contrast, the SCNCM90‐2AB particles maintained excellent structural integrity with no observable cracking (Figure 5g).^[^ ^31^ ^]^ HRTEM further identified extensive surface cracking and phase transformation features in cycled SCNCM90 particles (Figure 5b).^[^ ^32^ ^]^ HAADF‐STEM analysis demonstrated that in SCNCM90, electrolyte decomposition products progressively penetrated from the surface into the bulk, eroding the cathode‐electrolyte interface and inducing nanocrack formation through surface crystal structure (Figure 5c,d).^[^ ^33^ ^]^ This degradation process results in the formation of extensive *Fm*‐3*m* rock‐salt phase domains near the surface, which significantly hinder lithium‐ion diffusion and accelerate capacity fading.^[^ ^34^ ^]^ Although the *R*‐3*m* layered structures persist in the particle interior, geometric phase analysis (GPA) reveals widespread lattice deformation throughout the bulk (Figure 5d). Additionally, bulk kinks caused by stress concentration were observed in SCNCM90, which promoted crack propagation and particle delamination, as well as the stochastic nucleation of metastable O3‐type layered domains. These O3 phases are particularly vulnerable to oxygen loss, which facilitates TM ion migration from TM layers to Li layers, ultimately transforming the structure into an electrochemically inactive rock‐salt phase. These rock‐salt regions primarily formed near particle surfaces or crack interfaces due to sustained electrolyte exposure and enhanced oxygen loss in those areas (Figure 5e).^[^ ^35^, ^36^ ^]^ In sharp contrast, the SCNCM90‐2AB cathode exhibits significantly mitigated structural degradation, retaining a well‐ordered layered structure in the particle core with only slight surface disordering (Figure 5h,i). These observations highlight the dual functionality of the Al/B co‐modification strategy, which effectively suppresses the formation of an inactive NiO layer via surface stabilization and alleviation of lattice strain through subsurface cation disordering. STEM‐EELS provided further insights into oxygen stability. The O K‐edge spectrum of SCNCM90‐2AB exhibits a prominent pre‐edge peak at 530 eV, corresponding to well‐preserved O 2p‐TM 3d hybridization (Figure 5f,j). In comparison, SCNCM90 shows an attenuated pre‐edge signal, indicative of extensive oxygen vacancy formation. STEM‐EELS revealed progressive transition metal reduction from the bulk to the surface in SCNCM90, whereas reduction in SCNCM90‐2AB is confined to the surface.^[^ ^37^ ^]^ These findings confirm that Al/B co‐modification stabilizes lattice oxygen, suppresses bulk TM reduction, and maintains a more uniform electronic structure, thereby preventing capacity‐fading surface reconstructions.

To assess the mechanical and electrochemical stability of the cathodes, COMSOL Multiphysics simulations were conducted to model Li⁺ concentration distributions and interfacial stress evolution during cycling. For SCNCM90, the simulations reveal a marked decrease in lithium‐ion diffusion coefficients after 200 cycles, leading to pronounced Li^+^ concentration gradients and inhomogeneous ion distribution within the electrode (Figure S17, Supporting Information). This uneven Li⁺ flux, exacerbated by surface degradation, hindered efficient lithium transport within the bulk material. Prolonged cycling further intensified structural stress due to anisotropic volume changes associated with the H2‐H3 phase transition. Repetitive lattice expansion and contraction generate localized stress concentrations, accelerating mechanical failure (Figure S18, Supporting Information). In contrast, the SCNCM90‐2AB cathode exhibited a stabilized layered structure and improved lithium‐ion diffusion kinetics. This resulted in a more uniform Li⁺ distribution and substantially reduced stress accumulation during cycling. The simulations confirmed that the Al/B co‐modification strategy effectively suppresses microcrack formation, maintains mechanical integrity, and stabilizes the crystal framework under high‐voltage cycling conditions. Together, these experimental and computational findings underscore the critical role of Al/B co‐modification in addressing the structural and electrochemical challenges of Ni‐rich cathodes. By mitigating surface degradation, suppressing oxygen loss, and reducing internal stress accumulation, the dual‐modification strategy significantly enhances the long‐term cycling performance and durability of high‐energy lithium‐ion batteries.

## 3 Conclusion

In summary, the study proposed a dual‐modification strategy for single‐crystalline Ni‐rich SCNCM90 cathodes by integrating Al/B gradient co‐doping with an Al_5_BO_9_ surface coating to enhance structural and electrochemical stability. The Al/B gradient doping induced a lithium‐rich, vacancy‐disordered subsurface structure that effectively suppressed the H2‐H3 phase transition, alleviated cycling‐induced stress accumulation, and reinforced the bulk lattice framework. Concurrently, the chemically stable Al_5_BO_9_ coating mitigates detrimental electrode‐electrolyte interfacial reactions, further stabilizing the cathode surface. As a result, the dual‐modified SCNCM90 cathode exhibited outstanding electrochemical performance, achieving a capacity retention of 95.83% after 200 cycles at 4.5 V and maintaining 89.6% retention over 1000 cycles in a pouch‐type full cell. Moreover, the modified cathode demonstrates superior Li⁺ diffusion kinetics and thermodynamic stability throughout prolonged operation. Importantly, this dual‐modification strategy is applicable beyond SCNCM90 and holds great potential for another high‐performance cathode, including lithium cobalt oxide and lithium‐rich layered oxides. By addressing critical challenges associated with structural degradation and interfacial instability, this approach offers a promising pathway to extend the cycle life and energy density of next‐generation lithium‐ion batteries.

## Conflict of Interest

The authors declare no conflict of interest.

## Supporting information



Supporting Information

## Data Availability

The data that support the findings of this study are available from the corresponding author upon reasonable request.
