# Supplementary material for: Constructing Stable Sub/Surface Structure to Boost Superior Cyclabilities of Single‐Crystalline Ni‐Rich Cathode
Source: Adv Sci (Weinh). 2025 Aug 30;12(44):e10817. doi: 10.1002/advs.202510817 (PMC12667453; doi:10.1002/advs.202510817)
Supplement: Supplementary file 1 — Supporting Information [file ADVS-12-e10817-s001.docx]

**Supporting Information**

**Constructing Stable Sub/Surface Structure to Boost Superior Cyclabilities of Single-Crystalline Ni-rich Cathode**

Youqi Chu ^a, b, 1^, Kai Wang ^c, 1^, Changdong Chen ^a, 1^, Dixing Ni ^d,^ *, Qimeng Zhang ^a^, Hao Wang ^a^, Fan Peng ^a^, Anjie Lai ^a^, Gemeng Liang ^b,^ *, Chenghao Yang ^a,^ *

^a^ Guangzhou Key Laboratory for Surface Chemistry of Energy Materials, New Energy Research Institute, School of Environment and Energy, South China University of Technology, Guangzhou 510006, P. R. China

^b^ School of Chemical Engineering, The University of Adelaide, Adelaide, SA, 5000, Australia

^c^ School of Advanced Materials, Peking University, Shenzhen Graduate School, Shenzhen 518055, Guangdong, China.

^d^ High Performance Computing Department, National Supercomputing Center in Shenzhen, Shenzhen, Guangdong 518055, China.

^1^ These authors contributed equally to this work.

* Corresponding authors. E-mail addresses: nidx@nsccsz.cn (D. Ni), gemeng.liang@adelaide.edu.au (G. Liang), esyangc@scut.edu.cn (C. Yang)

**Experimental Section**

**Synthesis of Al/B Co-modified SCNCM90:** Commercial LiNi_0.9_Co_0.05_Mn_0.05_O_2_ (SCNCM90, 10 g) was dispersed in 50 mL of ethanol solution containing stoichiometric amounts of Al(NO_3_)_3_·9H_2_O and H_3_BO_3_, to obtain 1 mol%, 2 mol%, and 3 mol% Al/B-doped and Al_5_BO_9_-coated SCNCM90 samples. The mixture was stirred at 70 °C for 3 hours, followed by drying at 80°C for 12 hours. The resulting powders were then calcined at 600°C in air for 5 hours to yield the Al/B co-modified samples, denoted as SCNCM90-1AB, SCNCM90-2AB, and SCNCM90-3AB, respectively.

**Materials characterization:** Powder X-ray diffraction (XRD) was performed on a Bruker D8 Advance diffractometer (Cu-Kα, λ = 1.5418 Å) equipped with a LynxEye 1D detector, operating at 40 kV and 40 mA, with a step size of 0.02^o^ and a scan time of 0.8 s per step. Morphology was examined using a Sigma 300 field emission scanning electron microscope (FESEM). High-resolution structural imaging was conducted using a Titan Themis G2 HAADF-STEM and a Talos F200X microscope operating at 300 kV. Cryogenic transmission electron microscopy (Cryo-TEM) was carried out on a Titan Krios G3i at 300 kV with a Falcon 3 Direct Electron Detector. Post-cycling samples were collected in an Ar-filled glovebox, loaded on copper grids, and rapidly frozen in liquid nitrogen. Time-of-flight secondary ion mass spectrometry (ToF-SIMS) was performed on a PHI nanoTOF II instrument (analysis area: 200 μm × 200 μm, energy: 1 kV, current: 60 nA). X-ray absorption fine structure (XAFS) spectroscopy was conducted using a RapidXAFS 2M (Anhui Absorption Spectroscopy Analysis Instrument Co., Ltd.) in transmission mode at 20 kV and 20 mA with a Si (551) monochromator.

**Electrode fabrication and electrochemical testing:** The working electrodes were prepared by mixing SCNCM90 or Al/B co-modified SCNCM90 (85 wt%) with PVDF5130 (5 wt%) and Super P (10 wt%) in NMP solvent to form a uniform slurry. The slurry was coated on aluminum foil and vacuum-dried at 100 ^o^C for 24 hours. Electrode assembly was performed in an Ar-filled glovebox (O_2_ and H_2_O < 0.01 ppm). CR2025-type coin cells were assembled using lithium foil as the counter/reference electrode, and a 1.2 M LiPF_6_ solution in ethylene carbonate (EC) and dimethyl carbonate (DEC) (3:7 w/w) as the electrolyte. The cells were subjected to galvanostatic charge/discharge testing at various current rates using a LAND battery test system.

**Density functional theory (DFT) Calculations:** Spin-polarized DFT calculations were performed using the Vienna Ab initio Simulation Package (VASP) within the generalized gradient approximation (GGA) using the Perdew-Burke-Ernzerhof (PBE) functional. The projector augmented wave (PAW) method was employed to represent ionic cores, with a kinetic energy cutoff of 500 eV. Partial occupancies were treated using the Gaussian smearing method (smearing width: 0.05 eV). Electronic self-consistency was achieved at an energy threshold of 10^-5^ eV, and geometry optimization was considered converged when the maximum energy change per step was below 0.03 eV Å^-1^.

**COMSOL simulation:** Finite element simulations were performed using COMSOL Multiphysics to model lithium-ion diffusion and diffusion-induced stress in SCNCM90 and SCNCM90-2AB cathodes. The simulations were based on Fick's laws for Li^+^ transport and Hooke’s law for stress-strain relationships. Shrinkage of the cathode during charging and expansion during discharging were considered to evaluate mechanical deformation and internal stress evolution during cycling.


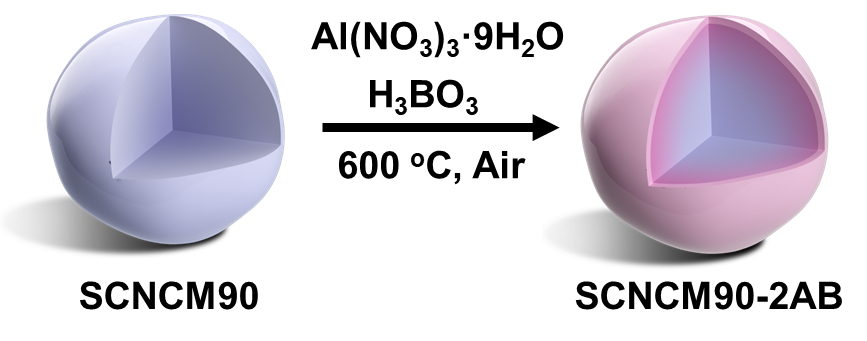


**Figure S1.** (a) Schematic illustration of the preparation process for SCNCM90-2AB cathode electrode.


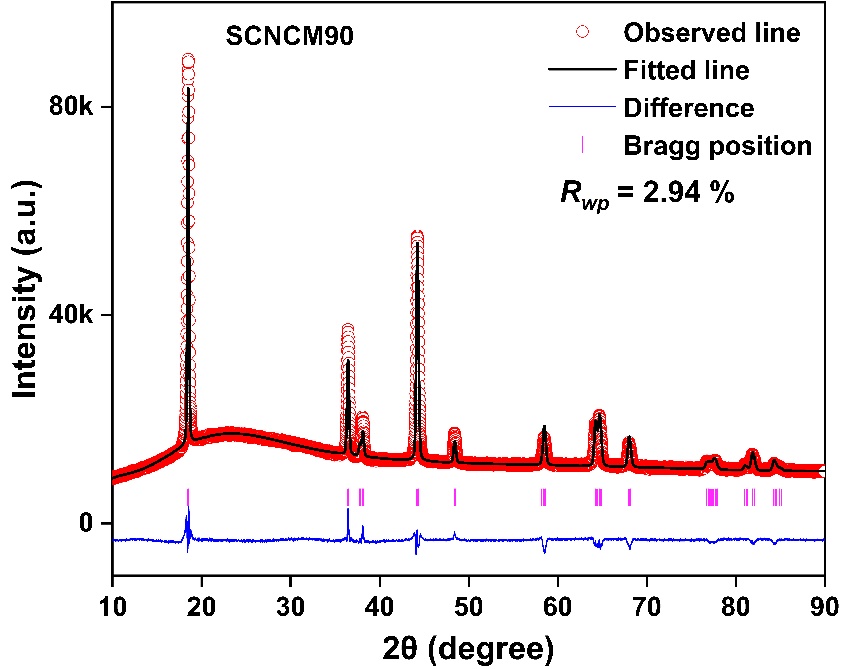


**Figure S2.** XRD Rietveld refinement results of SCNCM90.


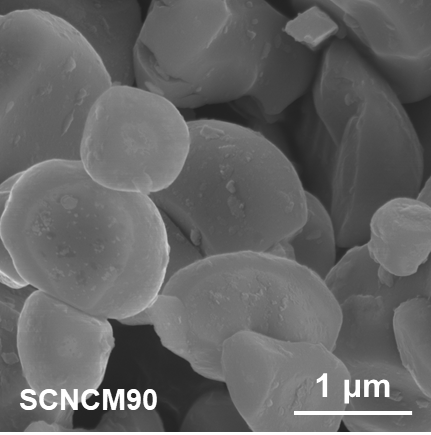


**Figure S3.** SEM images of SCNCM90.


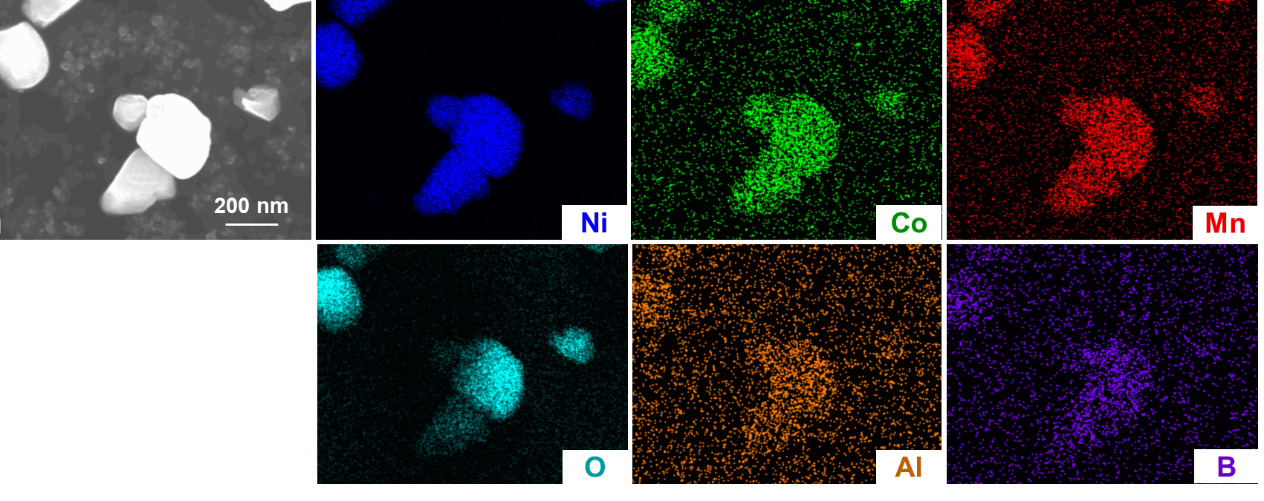


**Figure S4.** Elemental mapping of the SCNCM90-2AB.


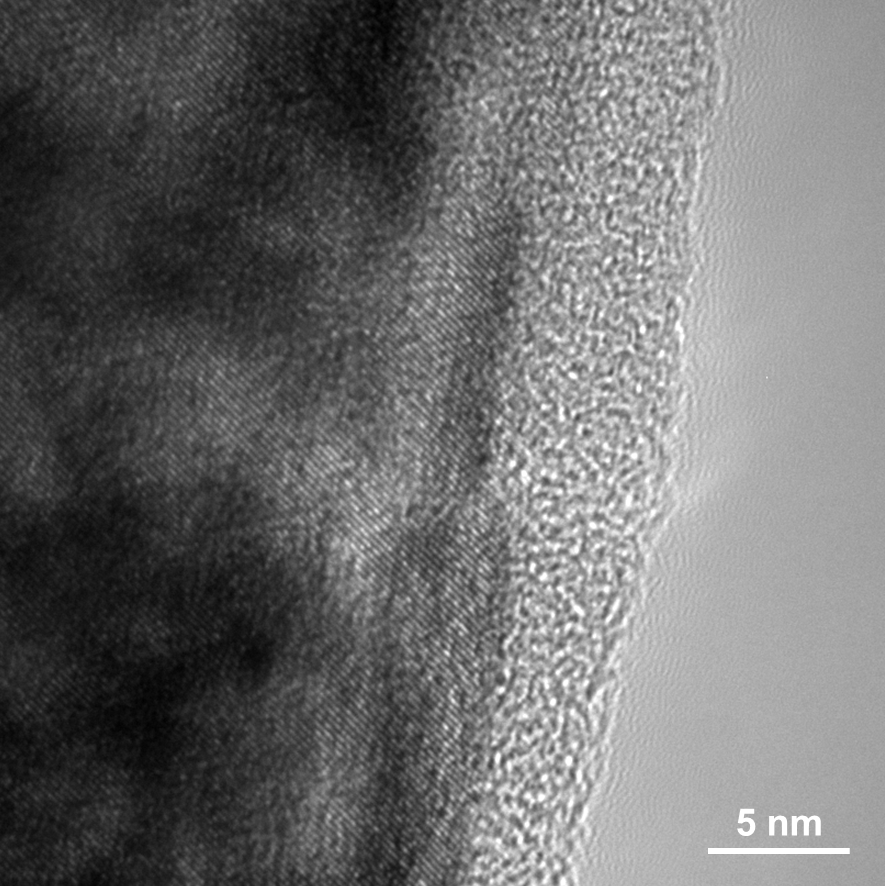


**Figure S5.** HRTEM image of SCNCM90-2AB.


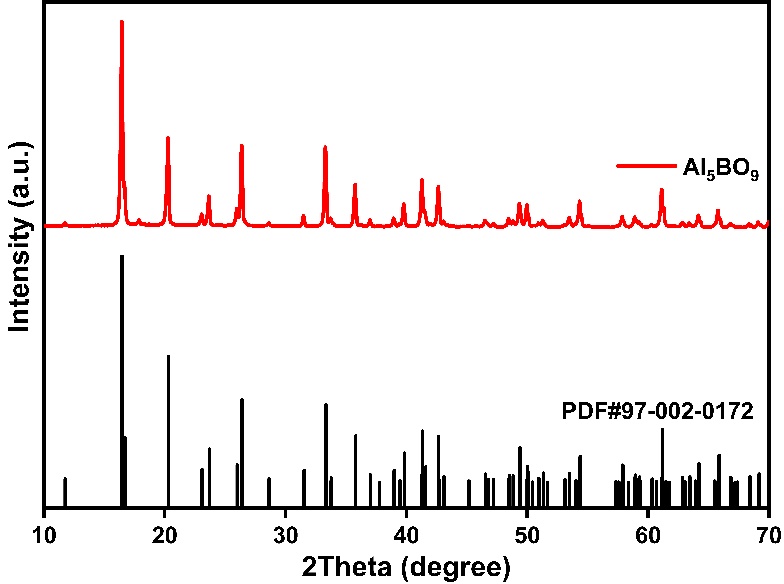


**Figure S6.** XRD patterns of Al_5_BO_9_ synthesized by the same process without SCNCM90 materials.


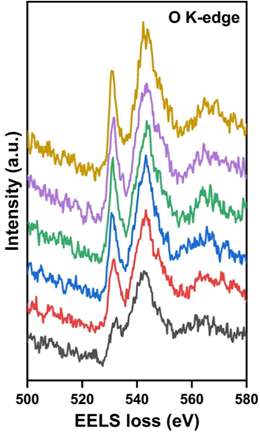


**Figure S7.** EELS spectra of the O K-edges extracted from the surface to the inside region of SCNCM90.


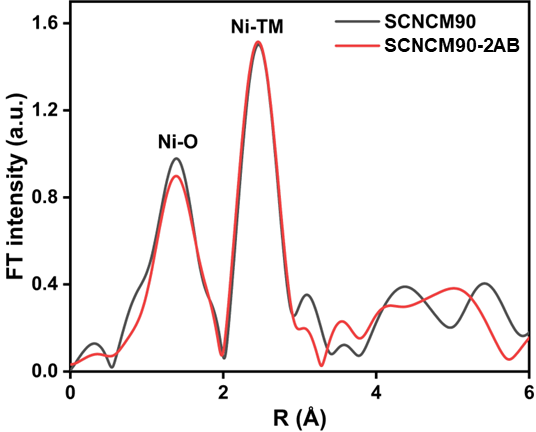


**Figure S8.** Ni K-edge of EXAFS R space curves for SCNCM90 and SCNCM90-2AB cathodes.


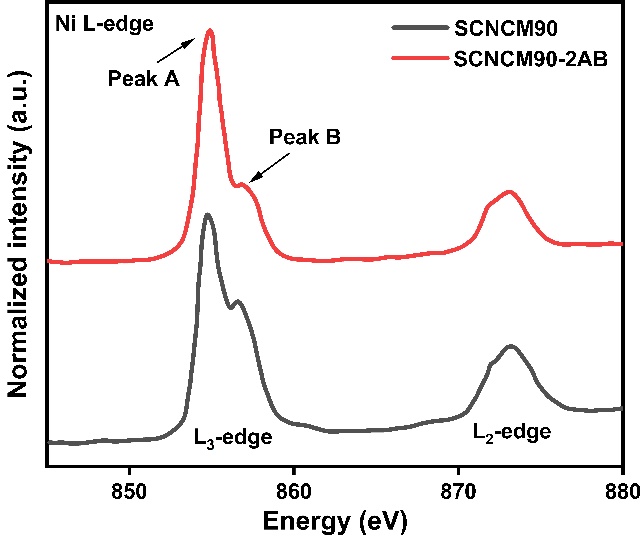


**Figure S9** Ni L-edge sXAS of SCNCM90 and SCNCM90-2AB.


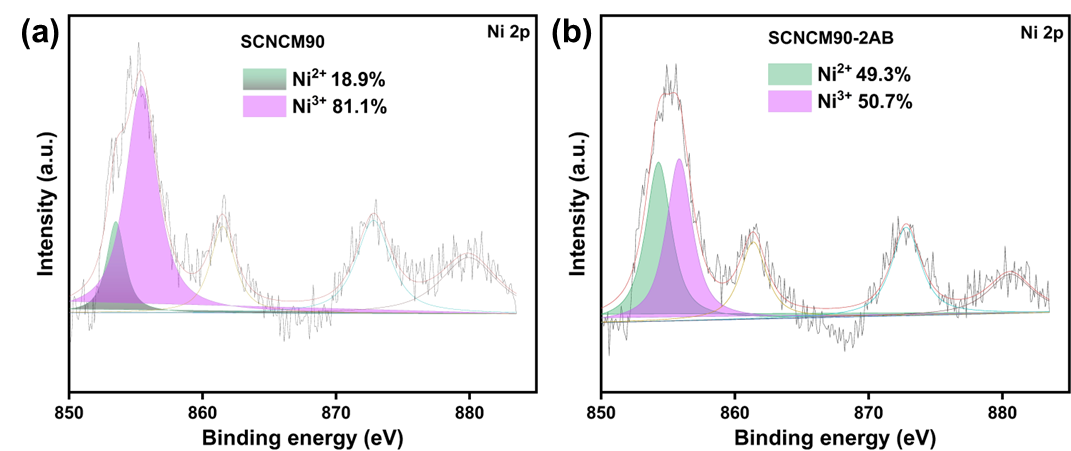


**Figure S10.** XPS spectra of SCNCM90 and SCNCM90-2AB for Ni 2p.


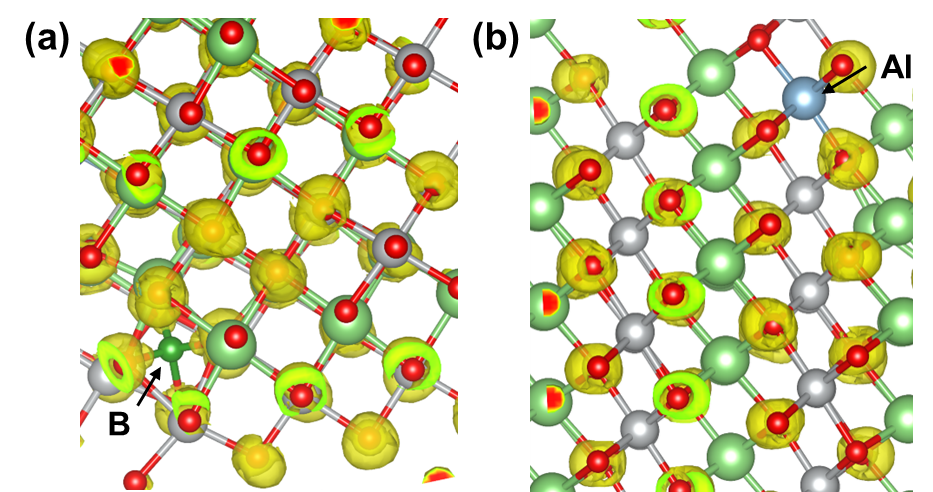


**Figure S11.** Charge difference of the (a) B-doped SCNCM90, (b) the Al-doped SCNCM90.


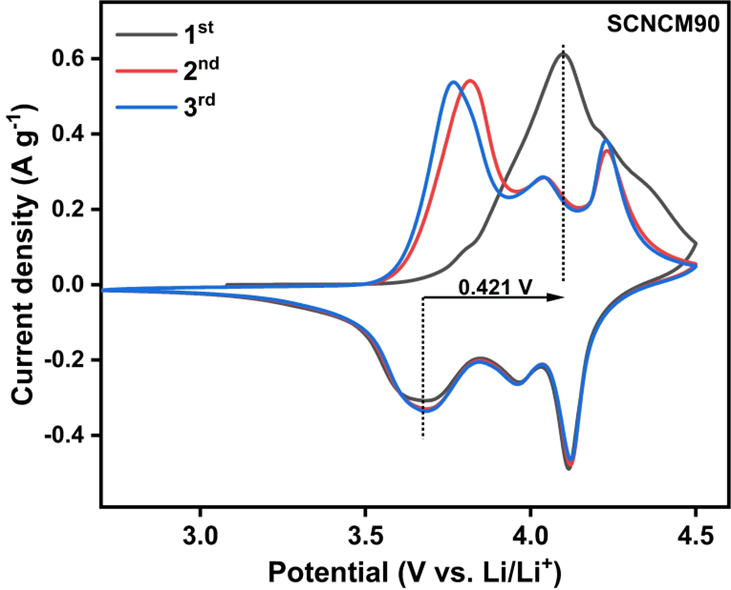


**Figure S12.** The cycle voltammetry curves of the first three cycle of SCNCM90 at a scan rate of 0.1 mV s^-1^.


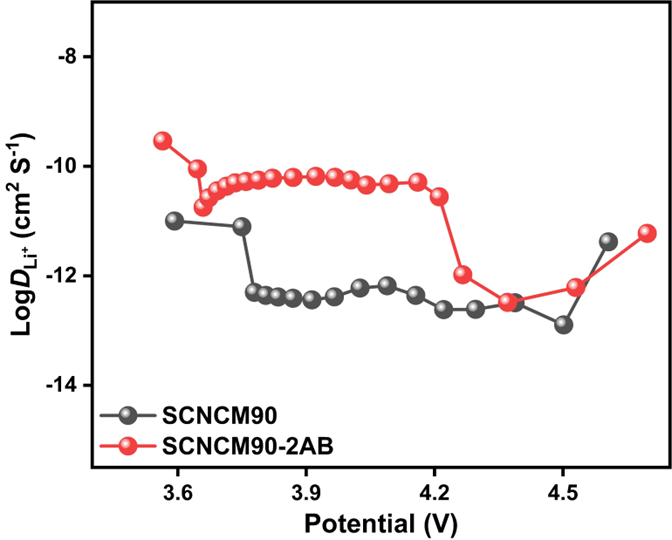


**Figure S13.** The Li^+^ diffusion coefficients of SCNCM90 and SCNCM90-2AB of GITT after 50 cycles.


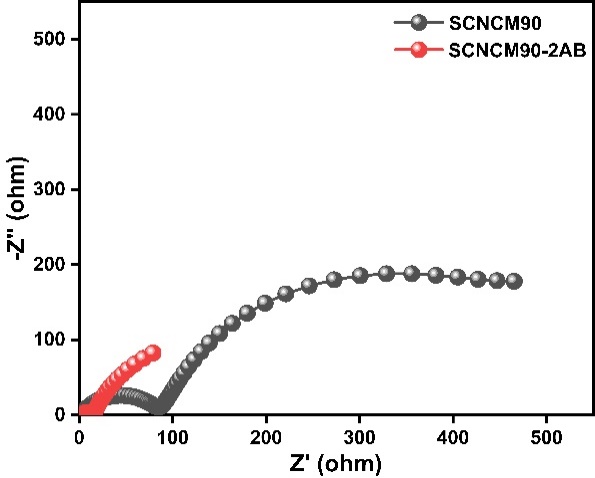


**Figure S14.** Nyquist plots of electrochemical impedances at the fully charged state (4.5 V) after 100 cycles for the SCNCM90 and SCNCM90-2AB.


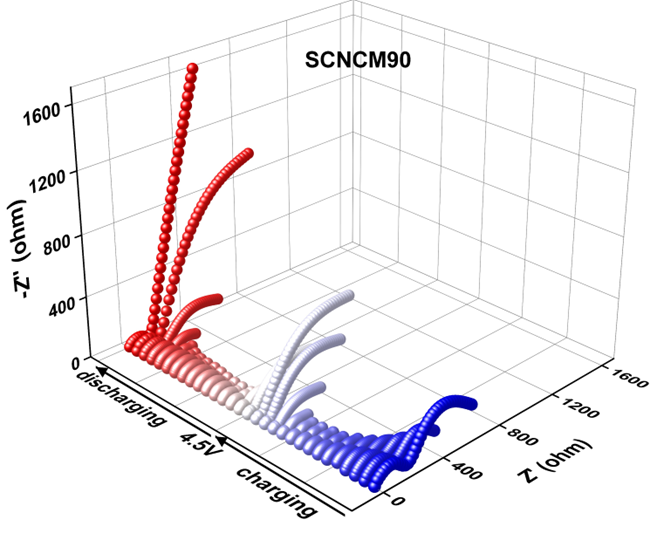


**Figure S15.** *In situ* EIS of SCNCM90 during the initial lithiation/delithiation process.


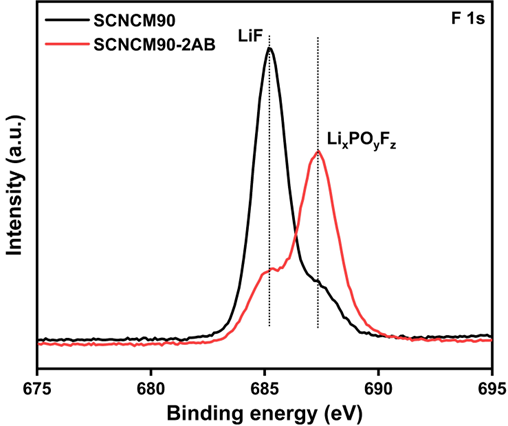


**Figure S16.** The XPS F 1s changes of SCNCM90 and SCNCM90-2AB during the 200 cycles.


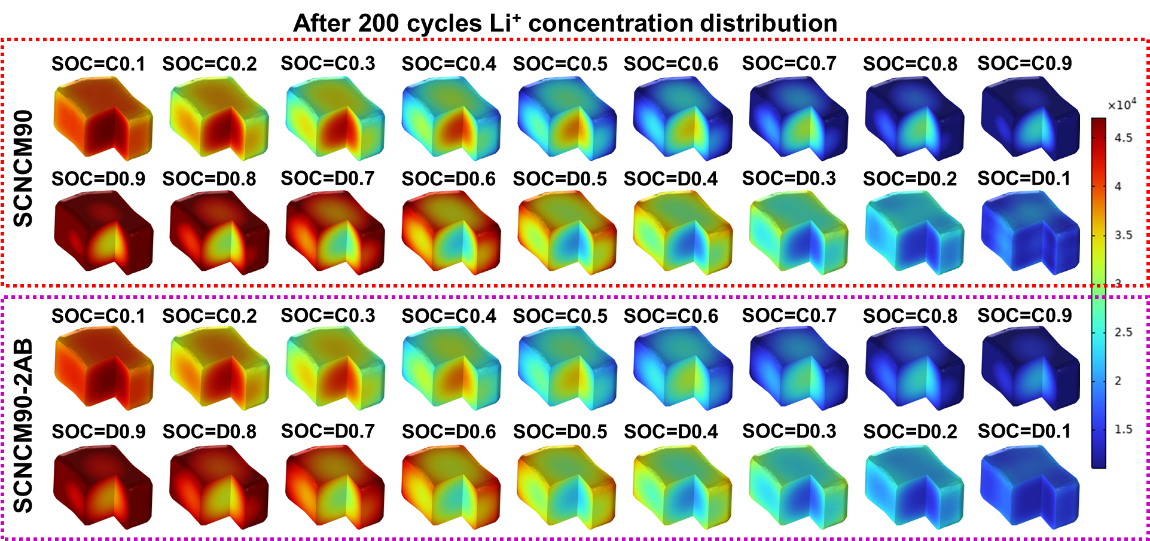


**Figure S17.** The simulation diagram of Li^+^ concentration distribution diagram at the SCNCM90 and SCNCM90-2AB surface interface with COMSOL Multiphysics after 200 cycles.


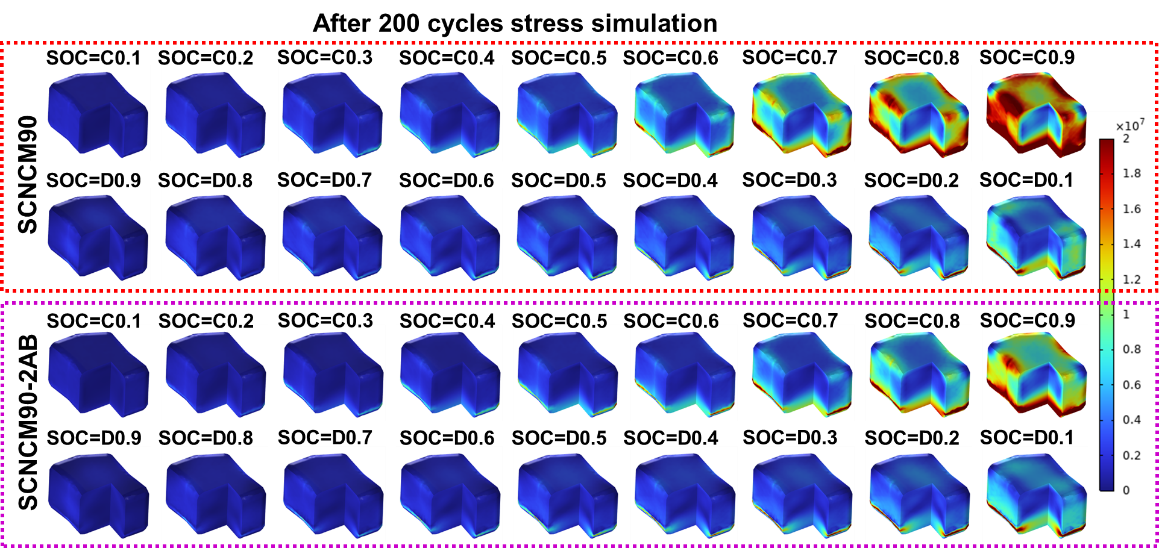


**Figure S18.** The stress simulation diagram at the SCNCM90 and SCNCM90-2AB surface interface with COMSOL Multiphysics after 200 cycles.

**Table S1.** ICP results for SCNCM90 and SCNCM90-AB samples (at%).

|  | Ni | Co | Mn | Al | B |
| --- | --- | --- | --- | --- | --- |
| SCNCM90 | 0.899 | 0.507 | 0.502 | / | / |
| SCNCM90-1AB | 0.898 | 0.509 | 0.504 | 0.093 | 0.021 |
| SCNCM90-2AB | 0.901 | 0.496 | 0.503 | 0.1937 | 0.045 |
| SCNCM90-3AB | 0.903 | 0.494 | 0.501 | 0.2977 | 0.063 |

**Table S2.** The refinement of SCNCM90 and SCNCM90-2AB.

| Sample | Li/Ni cation mixing | R_wp_ | R_p_ |
| --- | --- | --- | --- |
| SCNCM90 | 1.16% | 2.94% | 2.73% |
| SCNCM90-2AB | 1.21% | 2.01% | 2.14% |

**Table S3.** ICP results for SCNCM90 and SCNCM90-2AB samples after 200 cycles (mg L^-1^).

|  | Ni | Co | Mn |
| --- | --- | --- | --- |
| SCNCM90 | 178.3 | 37.6 | 28.9 |
| SCNCM90-2AB | 35.8 | 3.78 | 2.96 |
